# Study on the Constitutive Modeling of (2.5 vol%TiB + 2.5 vol%TiC)/TC4 Composites under Hot Compression Conditions

**DOI:** 10.3390/ma17030619

**Published:** 2024-01-27

**Authors:** Kehao Qiang, Shisong Wang, Haowen Wang, Zhulin Zeng, Liangzhao Qi

**Affiliations:** 1 College of Materials and Chemistry and Chemical Engineering, Chengdu University of Technology, Chengdu 610059, China; q1430347908@outlook.com; 2 College of Nuclear Technology and Automation Engineering, Chengdu University of Technology, Chengdu 610059, China; wsscdp@outlook.com; 3 School of Mechanical and Electrical Engineering, Chengdu University of Technology, Chengdu 610059, China; wdyx_teq@outlook.com (H.W.); zengzhulin1212@outlook.com (Z.Z.)

**Keywords:** titanium matrix composites, hot working, flow stress, constitutive model

## Abstract

The hot deformation behavior of titanium matrix composites plays a crucial role in determining the performance of the formed components. Therefore, it is significant to establish an accurate constitutive relationship between material deformation parameters and flow stress. In this study, hot compression experiments were conducted on a (2.5 vol%TiB + 2.5 vol%TiC)/TC4. The experiments were performed under temperatures ranging from 1013.15 to 1133.15 K and strain rates ranging from 0.001 to 0.1 s^−1^. Based on the stress–strain data obtained from the experiment, the constitutive models were established by using the Arrhenius model and the BP neural network algorithm, respectively. Considering the relationship between strain rate, hot working temperature, and flow stress, a comparative analysis was conducted to evaluate the prediction accuracy of two different constitutive models. The research results indicate that the flow stress of (2.5 vol%TiB + 2.5 vol%TiC)/TC4 increases with decreasing temperature and increasing strain rate, and the stress–strain curve shows obvious work hardening and softening behaviors. Both the Arrhenius model and the BP neural network algorithm are effective in predicting the hot compression flow stress of (2.5 vol%TiB + 2.5 vol%TiC)/TC4, but the average relative error and root mean square error of the BP neural network algorithm are smaller and the correlation coefficient is higher, thus possessing higher accuracy and reliability.

## 1. Introduction

Titanium matrix composites (TMCs) are extensively utilized in the aerospace and automotive industries due to their exceptional corrosion resistance, high temperature resistance, and mechanical properties [1,2,3]. (TiB + TiC)/TC4 is a typical TMC, and relevant studies [4] have indicated that the composite has the best combination of strength, modulus, and plasticity by introducing TiB and TiC simultaneously. Components made from (TiB + TiC)/TC4 typically undergo complicated casting and forging processes where the hot deformation process impacts the microstructural evolution and flow stress behavior of the material, consequently affecting the mechanical properties of the final formed component [5,6,7,8]. Therefore, it is essential to comprehend the influence of hot deformation parameters on microstructure evolution and flow stress for optimizing the hot working technology.

In the hot deformation process of metallic materials, the strain rate and hot working temperature are important factors that affect the material deformation behavior. Li et al. [9] investigated the deformation behavior of the TC18 alloy under various strain rates and hot working temperatures, analyzing the deformation behavior of the α and β phases. Cui et al. [10] conducted hot compression experiments to analyze the impact of different hot working conditions on flow stress of the TB6 alloy. Dan et al. [11] investigated the hot deformation behavior of the TC11 alloy and pointed out that the microstructure evolution under low strain rates was dominated by lamellar tissue spheroidization. Furthermore, several studies [12,13,14] have indicated the substantial influence of strain rate and hot working temperature on microstructure evolution and flow stress. Consequently, it is imperative to predict the hot deformation behavior of TMCs accurately under different strain rates and hot working temperatures.

Constitutive modeling is an effective mathematical modeling method for reflecting material flow stress behavior that has been widely applied in metallic materials [15,16,17]. According to the intrinsic mechanism of the model, constitutive models can be classified into phenomenological models, empirical models, and deep learning algorithm models. While phenomenological models have inherent physical meanings, empirical models and deep learning algorithm models are simpler and more suitable for intuitively analyzing the influence of strain rate and hot working temperature on the flow stress of metallic materials [18,19,20]. For example, Deb et al. [21] utilized a modified J-C model and deep learning algorithm to develop a constitutive model for TC4 alloy, which effectively predicted the flow stress behavior under various hot working conditions. Yang et al. [22] used the Arrhenius model and the BP neural network algorithm to accurately describe the constitutive relationship between strain rate, hot working temperature, and flow stress of the 7075 alloy, and they analyzed the prediction accuracy of these two models. Yu et al. [23] utilized empirical models and deep learning algorithm models to develop multiple sets of constitutive models for the TG6 alloy, and they compared the applicability of different models. The above studies indicate that both empirical models and deep learning algorithm models are effective in predicting the flow stress of metallic materials under different hot working conditions.

In this study, constitutive models for (2.5 vol%TiB + 2.5 vol%TiC)/TC4 were established based on the Arrhenius model and BP neural network algorithm, which aim to accurately predict the relationship between strain rate, hot working temperature, and flow stress. The subsequent research contents are as follows: Section 2 plots the stress–strain curves of (2.5 vol%TiB + 2.5 vol%TiC)/TC4 through hot compression experiments and analyzes the deformation behavior. Section 3 presents the constitutive models considering strain rate, hot deformation temperature, and flow stress of the TMCs by using the Arrhenius model and BP neural network algorithm, respectively. Section 4 compares the prediction accuracy of two models. Section 5 provides a summary and conclusions.

## 2. Experimental Methods and Results

**Figure 1:**
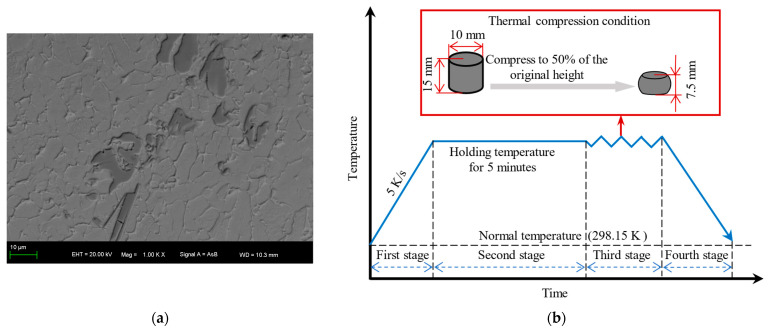
Experiment methods: (**a**) initial microstructure; (**b**) experimental process.

The TMC used in this study is (2.5 vol%TiB + 2.5 vol%TiC)/TC4; both TiB and TiC particles have a volume fraction of 2.5%. The initial microstructure of the sample is shown in Figure 1a. The matrix material used in this study is TC4 titanium alloy, which consists of an α phase and an intergranular β phase. The second-phase particles are TiB and TiC, in which TiB has a lamellar shape and TiC has an equiaxial shape. Figure 1b illustrates the specific hot compression experiment, which is divided into four stages according to the relevant references [5,6,7]. Firstly, the specimen is heated from the normal temperature to the hot compression temperature. Subsequently, keep the specimen warm for 5 min to avoid temperature deviations. In the third stage, the specimen is compressed from the original height (15 mm) to 50% (7.5 mm) with a specified strain rate, corresponding to a true strain of 0.7. Finally, the specimen is cooled to a normal temperature in the air for observation. The hot working temperature and strain rate conditions for the experiment are listed in Table 1. The stress–strain behavior of the TMCs in the two-phase region is studied in this paper. Therefore, the hot working temperature should be less than the temperature of the phase transition point [24].

**Figure 2:**
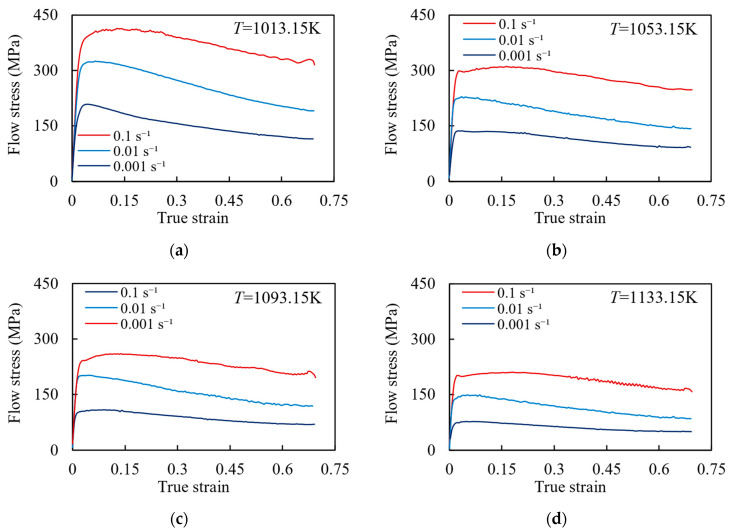
Stress–strain curves under different temperatures: (**a**) 1013.15 K; (**b**) 1053.15 K; (**c**) 1093.15 K; (**d**) 1133.15 K.

The Gleeble 3500 was used for the hot compression experiment. To ensure the validity of the experiment results, two experiments were conducted under each experiment condition, and the stress–strain curve error of the two experiments was ensured to be less than 5%. Meanwhile, according to [25,26], the influence of temperature and friction coefficient on the stress–strain curve was modified. The true stress–strain curves of the specimen under different strain rates and temperatures are shown in Figure 2. It shows that with the temperature increasing and the strain rate decreasing, the flow stress decreases. Additionally, the flow stress curves under all conditions have work hardening, dynamic recovery (DRV), and dynamic recrystallization (DRX) behaviors. In the initial deformation stage, a significant amount of dislocation proliferation and accumulation occurs, resulting in a rapid increase and peak in flow stress. This behavior is known as work hardening. As strain increases, the softening effect caused by the DRV process strengthens gradually. Simultaneously, internal energy accumulates in the composites, leading to the nucleation and growth of recrystallized grains, which triggers the DRX process and further enhances the softening effect. As the DRV and DRX processes progress, the work hardening effect gradually weakens, resulting in a gradual decrease in flow stress and a tendency towards stabilization (Note: some data in Figure 2 have been used in our previous study [27]).

**Figure 3:**
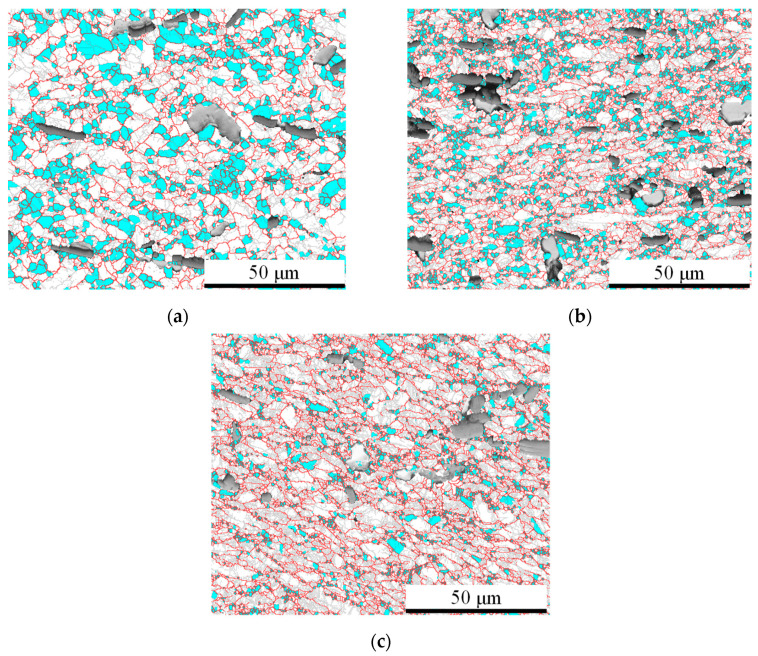
EBSD analysis results under the temperature of 1053.15 K and different strain rates: (**a**) 0.001 s^−1^, (**b**) 0.01 s^−1^, and (**c**) 0.1 s^−1^.

The results of electron back scatter diffraction (EBSD) under different strain rates are shown in Figure 3. It can be seen that as the strain rate decreases, the fraction and grain sizes of DRX increase significantly. This is because the higher strain rate provides a greater driving force for the DRX process, resulting in a faster DRX nucleation rate. Simultaneously, the high strain rate hinders the growth of DRX grains, forming refined equiaxed DRX grains. Therefore, a decrease in strain rate leads to an increase in both the fraction and grain size of DRX.

**Figure 4:**
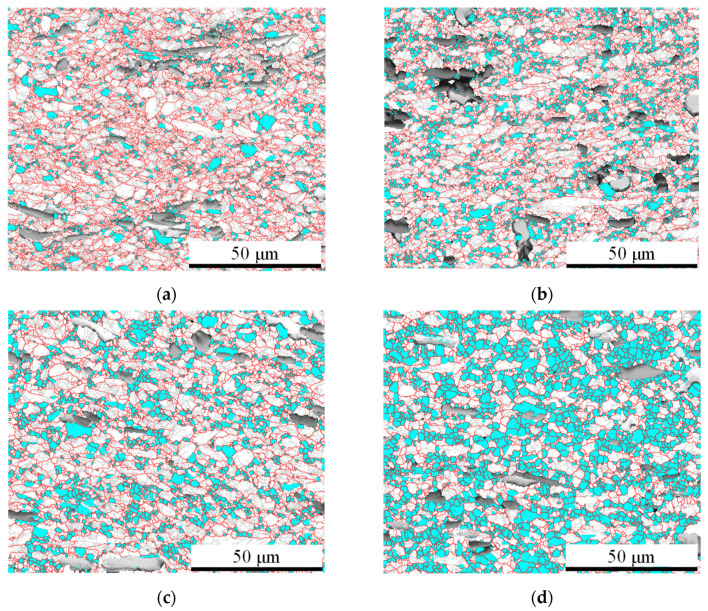
EBSD analysis results under the strain rate of 0.01 s^−1^ and different temperatures: (**a**) 1013.15 K, (**b**) 1053.15 K, (**c**) 1093.15 K, and (**d**) 1133.15 K.

Figure 4 shows the EBSD results under different temperatures. Contrary to the effect of strain rate, an increase in temperature leads to an increase in the DRX fraction. This is due to the nucleation and growth processes of DRX grains being influenced by thermodynamics and higher temperatures promoting dislocation evolution and grain boundary migration. Consequently, this leads to a significant increase in the nucleation rate and size of DRX grains.

## 3. Description of Constitutive Model

In this section, the Arrhenius model and the BP neural network algorithm were used to establish constitutive models for (2.5 vol%TiB + 2.5 vol%TiC)/TC4. The two constitutive models were compiled in MATLAB R2022b software to predict the flow stress under different temperatures, strain rates, and strains.

### 3.1. Arrhenius Model

The Arrhenius model, proposed by Sellars and Tegart [28], is an empirical constitutive model commonly used to describe the relationship between flow stress, strain rate, and deformation temperature during the hot deformation process of metal materials. It is widely utilized due to the minimal number of parameters and high accuracy. The mathematical expression for the Arrhenius model is as follows:

(1) $$\dot{\varepsilon }=AF(\sigma )\exp(-\frac{Q}{RT})$$

At different stress levels, *F*(*σ*) can be expressed by Equation (2):

(2) $$F\left(\sigma \right)=\left\{ \begin{array}{l} \sigma^{n}\;\;\alpha \sigma <0.8\\ \exp\left(\beta \sigma \right)\;\;\alpha \sigma >1.2\\ {\left[\sinh\left(\alpha \sigma \right)\right]}^{m}\;\;\mathrm{all}\;\sigma \; \end{array}\right.$$

where: *σ*, *ε*, and *T* represent the flow stress (MPa), strain rate (s^−1^), and hot working temperature (K), respectively; *R* and *Q* are the gas constant (8.314 J/(mol·K)) and deformation activation energy (kJ/mol), respectively; *A*, *α*, and *β* are material constants; *m* and *n* are the work hardening exponent; and *α* = *β*/*n*.

The following can be derived from Equations (1) and (2) by applying logarithms to both sides:

(3) $$\ln\dot{\varepsilon }=\ln A_{1}+n\ln\sigma -\frac{Q}{RT}$$

(4) $$\ln\dot{\varepsilon }=\ln A_{2}+\beta \sigma -\frac{Q}{RT}$$

(5) $$\ln\dot{\varepsilon }=\ln A_{3}-\frac{Q}{RT}+m\ln\left[\sinh\left(\alpha \sigma \right)\right]$$

In Equations (3) and (4), under the condition of constant temperature, a linear relationship exists between $\ln\dot{\varepsilon }$ and lnσ and between $\ln\dot{\varepsilon }$ and σ, whose slopes are denoted as *n* and *β*, respectively.

**Figure 5:**
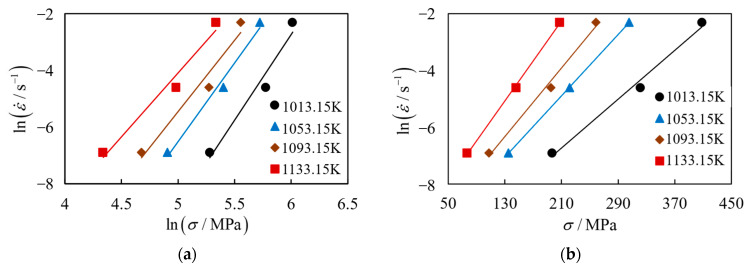
Linear relationship between stress and strain: (**a**) $\ln\dot{\varepsilon }$-lnσ; (**b**) $\ln\dot{\varepsilon }$-σ.

Taking *ε* = 0.1 as an example, Figure 5 shows the plots of $\ln\dot{\varepsilon }$-lnσ and $\ln\dot{\varepsilon }$-σ for (2.5 vol%TiB + 2.5 vol%TiC)/TC4 within the range of *ε* = 0.1 to 0.001 s^−1^. By calculating the average slope, the results are as follows: *n* = 5.2816, *β* = 0.0285; further, *α* = *β/n* = 0.005396.

Under the condition that 1/T is constant, both sides of Equation (5) take the partial derivative of $\dot{\varepsilon }$ and Equation (6) is obtained:

(6) $$m=\frac{\partial \ln\left[\sinh\left(\alpha \sigma \right)\right]}{\partial \ln\dot{\varepsilon }}$$

Under the condition that $\dot{\varepsilon }$ is constant, both sides of Equation (5) take the partial derivative of 1/T and Equation (7) is obtained:

(7) $$Q=Rm\frac{\partial \ln\left[\sinh\left(\alpha \sigma \right)\right]}{\partial \left(1/T\right)}$$

Assuming $b=\frac{\partial \ln\left[\sinh\left(\alpha \sigma \right)\right]}{\partial \left(1/T\right)}$, Equation (7) can be simplified as:

(8) Q=Rmb

**Figure 6:**
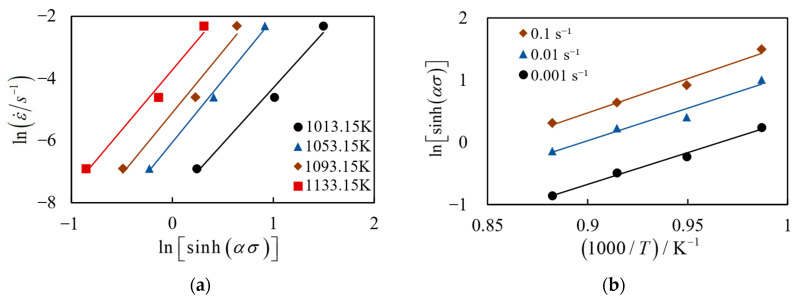
Linear relationship of stress–strain rate and stress–temperature: (**a**) $\ln\dot{\varepsilon }$-ln[sinh(ασ)]; (**b**) ln[sinh(ασ)]-1000/T.

By substituting α = 0.005396 into Equation (5), the linear relationship between $\ln\dot{\varepsilon }$-ln[sinh(ασ)] and ln[sinh(ασ)]-1000/T under constant temperature and constant stress can be obtained, whose slopes are expressed as *m* and *b*, respectively. Taking ε = 0.1 as an example, Figure 6 shows the plots of $\ln\dot{\varepsilon }$-ln[sinh(ασ)] and ln[sinh(ασ)]-1000/T for (2.5 vol%TiB + 2.5 vol%TiC)/TC4 composites within the range of *ε* = 0.1–0.001 s^−1^. By calculating the average slope, the results are as follows: *m* = 3.8622, *b* = 10.5647, and *Q* = 339.2360 kJ/mol.

According to relevant research, the influence of hot deformation temperature and strain rate on the hot deformation can be described by the temperature compensation deformation rate factor Z [29], which is expressed as:

(9) $$Z=\dot{\varepsilon }\exp\left(-\frac{Q}{RT}\right)=A{\left[\sinh\left(\alpha \sigma \right)\right]}^{n}$$

Both sides of Equation (9) take the logarithms to obtain Equation (10):

(10) lnZ=lnA+nln[sinh(ασ)]

**Figure 7:**
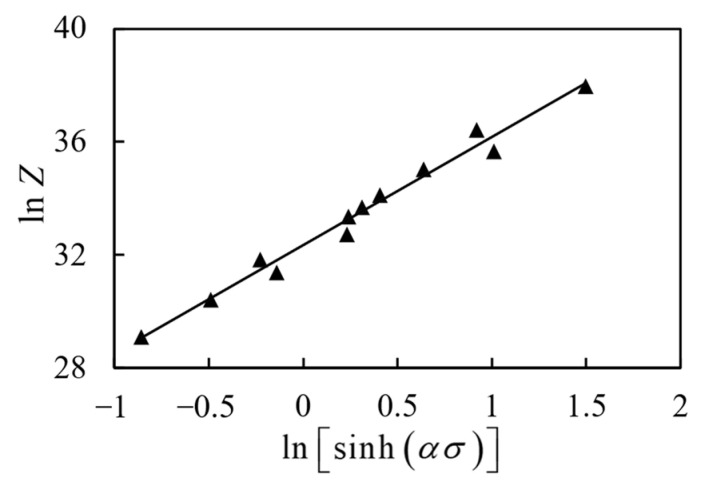
The linear relationship of lnZ-ln[sinh(ασ)].

As shown in Figure 7, a linear relationship between lnZ and ln[sinh(ασ)] can be obtained by combining the *Q* value, Equations (9) and (10). Obviously, the intercept in Figure 7 is lnA = 32.356.

Repeating the above solving process, with *ε* = 0.1 as the gradient, values of *α*, *m*, *Q*, and lnA for (2.5 vol%TiB + 2.5 vol%TiC)/TC4 within the strain range of 0.1 to 0.7 are calculated, respectively. The results are listed in Table 2.

**Figure 8:**
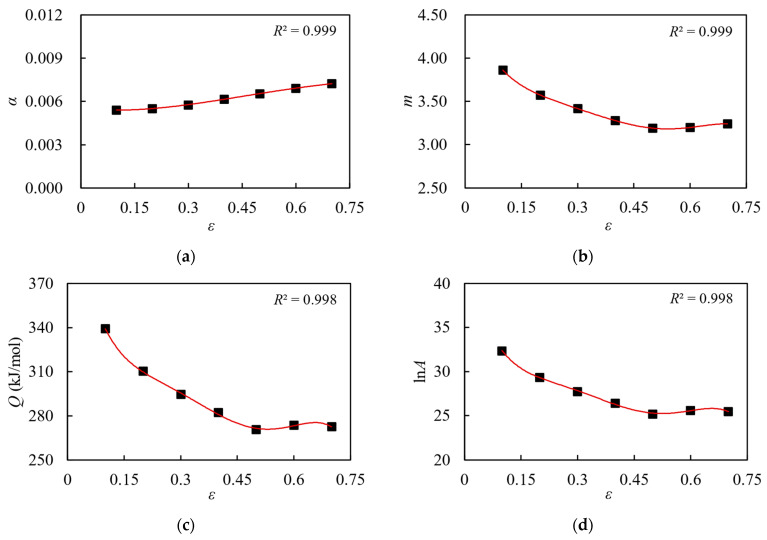
Fitting curves of different parameters with strain: (**a**) *α*-ε; (**b**) *m*-ε; (**c**) *Q*-ε; (**d**) lnA-ε.

Figure 8 shows the data for *α*, *m*, *Q*, and lnA under different strains. The change pattern between these parameters and strain is represented by a curve obtained through fifth-order polynomial fitting [30], and the specific expression is given by Equations (11)–(14). Figure 8 clearly illustrates that the fitted curve accurately reflects the variation law of each parameter and strain.

(11) $$\alpha =0.013\varepsilon^{5}-0.022\varepsilon^{4}+0.003\varepsilon^{3}+0.01\varepsilon^{2}-0.002\varepsilon +0.006$$

(12) $$m=-104.333\varepsilon^{5}+215.364\varepsilon^{4}-166.099\varepsilon^{3}+61.77\varepsilon^{2}-12.699\varepsilon +4.66$$

(13) $$Q=-16917.083\varepsilon^{5}+34067.992\varepsilon^{4}-25638.434\varepsilon^{3}+9144.104\varepsilon^{2}-1701.418\varepsilon +440.404$$

(14) $$\ln A=-1951.25\varepsilon^{5}+3910.417\varepsilon^{4}-2924.771\varepsilon^{3}+1033.121\varepsilon^{2}-188.512\varepsilon +43.436$$

Furthermore, by rearranging Equations (1) and (2), the constitutive relationship for (2.5 vol%TiB + 2.5 vol%TiC)/TC4 within the range of hot working temperature from 1013.15 to 1133.15 K and strain rate from 0.001 to 0.1 s^−1^ can be expressed as:

(15) $$\sigma =\frac{1}{\alpha }\ln\left\{{\left(\frac{\dot{\varepsilon }}{A}\exp\left(\frac{Q}{RT}\right)\right)}^{1/m}+{\left[{\left(\frac{\dot{\varepsilon }}{A}\exp\left(\frac{Q}{RT}\right)\right)}^{2/m}+1\right]}^{1/2}\right\}$$

**Figure 9:**
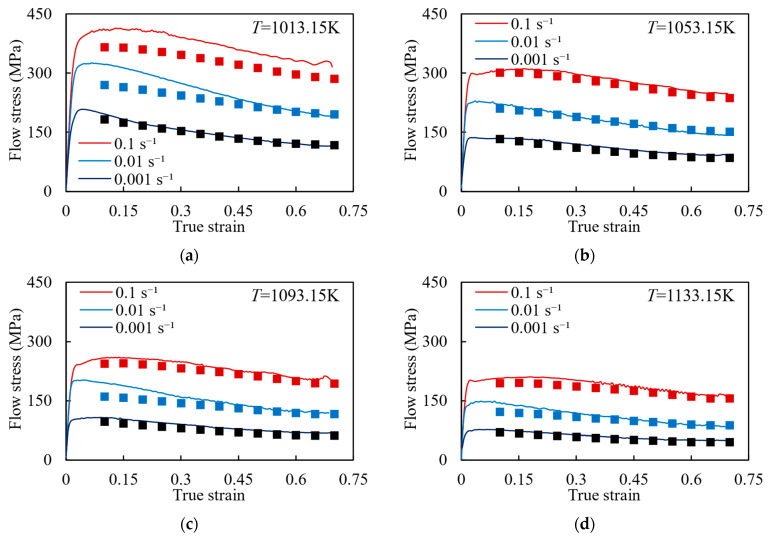
Comparison of stress–strain curves between experimental results (curves) and Arrhenius model prediction results (symbols): (**a**) 1013.15 K; (**b**) 1053.15 K; (**c**) 1093.15 K; and (**d**) 1133.15 K.

The α, *m*, *Q*, and *A* values under various strain levels are calculated using Equations (11)–(14) and then incorporated into Equation (15), from which the constitutive relationship between flow stress and strain in the (2.5 vol%TiB + 2.5 vol%TiC)/TC4 under different strain rates and hot working temperatures is obtained. As shown in Figure 9, the results obtained from the Arrhenius model are in good agreement with the experimental results under various temperatures and strain rates. It is suggested that the Arrhenius model is capable of accurately predicting the flow stress behavior of the (2.5 vol%TiB + 2.5 vol%TiC)/TC4 during the hot working process.

### 3.2. BP Neural Network Algorithm

In the process of establishing empirical or semi-empirical constitutive models for materials using the Arrhenius equation, numerical errors are introduced by numerical methods such as approximate solutions and linear regression fitting [31]. Therefore, deep learning algorithms have been increasingly utilized in constitutive modeling due to their non-linear modeling capabilities, adaptability, and generalization abilities [32,33]. The BP neural network algorithm, proposed by Rumelhart and McClelland [34], is widely recognized and extensively used. The BP neural network algorithm model comprises two processes: signal forward propagation and error back propagation, in which the weights and thresholds are adjusted iteratively. The training process concludes when the pre-set model training times are reached or the error is reduced to an acceptable level [35].

**Figure 10:**
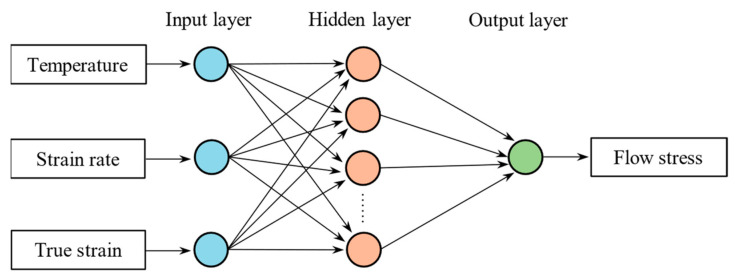
The BP neural network structure established in this study.

As shown in Figure 10, the structure of the BP neural network algorithm in this study includes an input layer, hidden layers, and an output layer. The input layer consists of three neurons that receive external data corresponding to deformation temperature, strain rate, and strain. The hidden layer is used for nonlinear transformation and feature extraction. In order to prevent overfitting, the hidden layer is designed as a single layer with 16 neurons, which is determined based on references [36]. The output layer, responsible for the final data output, consists of one neuron representing the flow stress.

In addition, due to the significant difference in data magnitude and range between temperature, strain rate, strain, and flow stress, it is necessary to use the linear normalization method. As indicated in Equations (16) and (17), normalization and denormalization of the input and output data are necessary in order to improve the computational speed, accuracy, and robustness of the BP neural network model:

(16) $$x^{\prime }=\left(x-x_{\min}\right)/\left(x_{\max}-x_{\min}\right)$$

(17) $$x=x^{\prime }\left(x_{\max}-x_{\min}\right)+x_{\min}$$

where x and $x^{\prime }$ are the data before and after normalization, respectively, and $x_{\max}$ and $x_{\min}$ are the maximum and minimum value, respectively, of the data before normalization.

**Figure 11:**
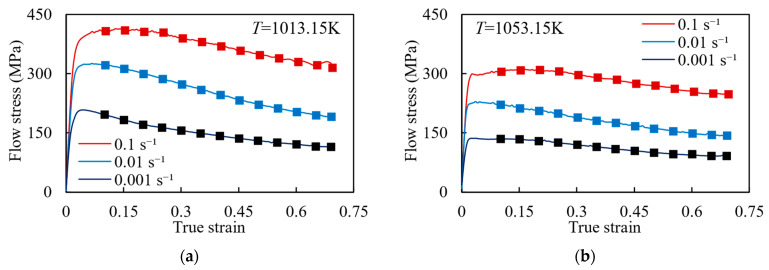

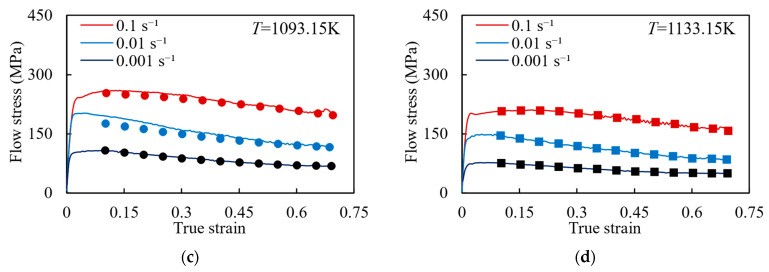
Comparison of stress–strain curves between experimental results (curves) and BP neural network prediction results (symbols), where 1093.15 K is the prediction result: (**a**) 1013.15 K; (**b**) 1053.15 K; (**c**) 1093.15 K; and (**d**) 1133.15 K.

The experimental data within the strain range of 0.1 to 0.7, with a gradient of 0.005, were used as data samples for a total of 1428 sets of data. Among these, data under deformation temperatures of 1013.15 K, 1053.15 K, and 1133.15 K were selected as the training set for the model, totaling 1071 sets of data; data under a temperature of 1093.15 K were used as the validation set, consisting of 357 sets of data. During the model training, the maximum number of iterations was set to 15,000, the learning rate to 0.1, and the convergence error to 0.0001. The transfer function for the hidden layer was set as the hyperbolic tangent function, while the transfer function for the output layer was set as the linear transfer function. Figure 11 shows the prediction results of the BP neural network algorithm. It is noteworthy that the stress–strain data of 1013.15 K, 1053.15 K, and 1133.15 K were used to model training, meaning the calculated results are highly consistent with the experimental results. However, the stress–strain data of 1093.15 K were only used to verify the validity of the BP neural network model instead of model training. Therefore, when analyzing the validity of this model, it is suggested to focus on Figure 11c. The BP neural network algorithm has a strong agreement with the experimental results under different hot working conditions, thereby validating the accuracy of the model established by the BP neural network algorithm.

## 4. Model Evaluation

To assess the accuracy of the above two models in predicting the hot deformation behaviors of (2.5 vol%TiB + 2.5 vol%TiC)/TC4, Equations (18)–(21) were used to calculate the relative error (*RE*), average absolute relative error (*AARE*), root mean square error (*RMSE*), and correlation coefficient (*R*), which provide a measure of the disparity between the predicted and experimental results:

(18) $$RE=\left|\frac{\sigma_{E}^{i}-\sigma_{T}^{i}}{\sigma_{T}^{i}}\right|\times 100\%$$

(19) $$AARE=\frac{1}{N}\sum_{i=1}^{N}\left|\frac{\sigma_{E}^{i}-\sigma_{T}^{i}}{\sigma_{T}^{i}}\right|\times 100\%$$

(20) $$RMSE=\sqrt{\frac{1}{N}\sum_{i=1}^{N}{\left(\sigma_{E}^{i}-\sigma_{T}^{i}\right)}^{2}}$$

(21) $$R=\frac{\sum_{i=1}^{N}\left(\sigma_{T}^{i}-{\bar{\sigma }}_{T}\right)\left(\sigma_{E}^{i}-{\bar{\sigma }}_{E}\right)}{\sqrt{\sum_{i=1}^{N}{\left(\sigma_{T}^{i}-{\bar{\sigma }}_{T}\right)}^{2}}\sqrt{\sum_{i=1}^{N}{\left(\sigma_{E}^{i}-{\bar{\sigma }}_{E}\right)}^{2}}}$$

where σ and $\bar{\sigma }$ represent true stress and mean value of true stress, respectively, and subscripts *E* and *T* represent the predicted values and experimental values, respectively; *N* represents the total number of samples, and *i* represents the *i*-th item in the samples (in each working condition, 119 groups of data in the strain range of 0.1 to 0.7 were selected for calculation with a gradient of 0.05).

**Figure 12:**
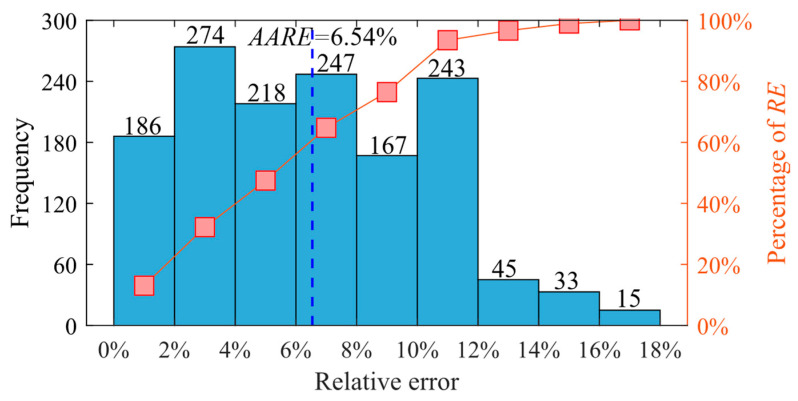
The relative error distribution of the Arrhenius model-predicted values.

Figure 12 displays the relative error distributions of the Arrhenius model-predicted values under four different hot working temperatures. The *AARE* value was calculated at 6.54%; it is noteworthy that 48.32% of the *RE* values are greater than 6.54% while 51.68% are less than 6.54%. It shows that the predicted values are in good agreement with the experimental values.

**Figure 13:**
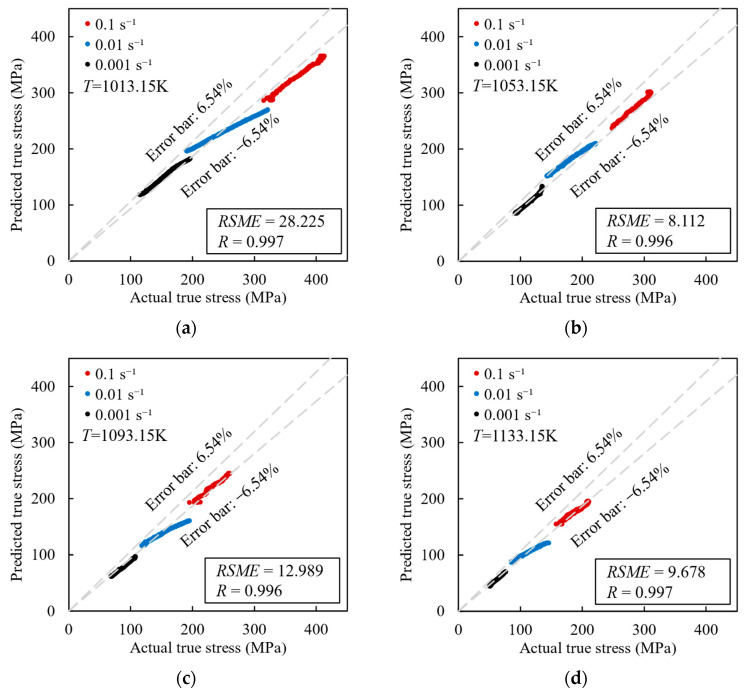
The correlation between Arrhenius model-predicted values and experimental values: (**a**) 1013.15 K; (**b**) 1053.15 K; (**c**) 1093.15 K; and (**d**) 1133.15 K.

Figure 13 shows the correlation between the predicted values and the experimental values. Most of the data fall within the mean relative error range of ±6.54%. The *RMSE* values under the four hot working temperatures are as follows: 28.225, 8.112, 12.989, and 9.678; the corresponding *R* values are 0.997, 0.996, 0.996, and 0.997, respectively. The overall *RMSE* and *R* values for the data are 16.769 and 0.994, respectively. These results indicate that the Arrhenius model has low prediction errors and significant correlations between the predicted values and experimental values. Therefore, the Arrhenius model effectively predicts the flow stress behavior of (2.5 vol%TiB + 2.5 vol%TiC)/TC4.

**Figure 14:**
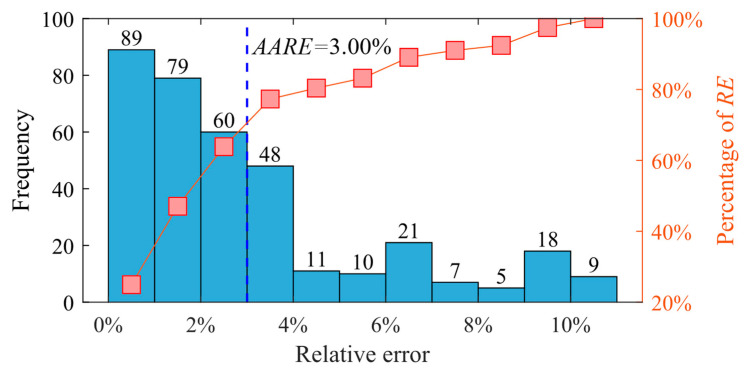
The relative error distribution of BP neural network algorithm prediction values.

Figure 14 shows the relative error distribution of the predicted values for the validation set (at 1093.15 K) of the BP neural network algorithm. The *AARE* value is 3.00%, with 36.13% of the *RE* values being greater than 3.00% and 63.87% being less than 3.00%. These findings suggest a strong correlation between the predicted values and the experimental values.

**Figure 15:**
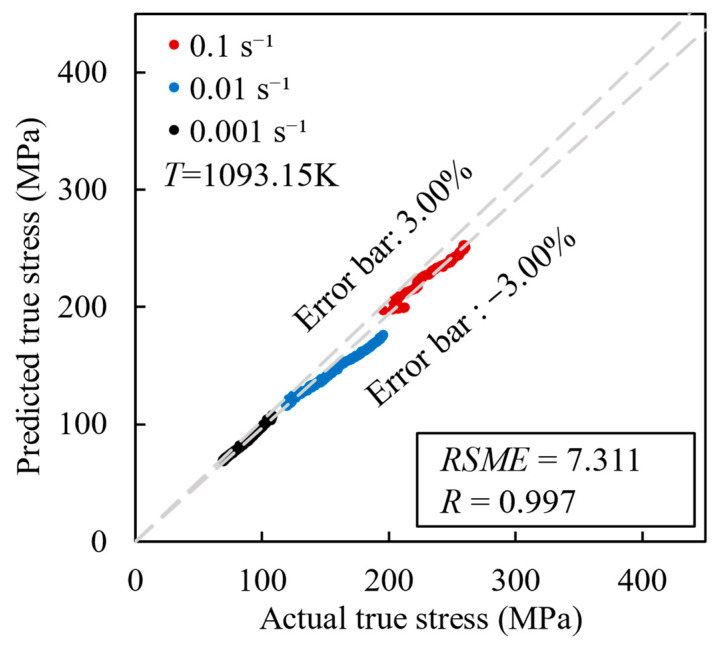
Correlation between BP neural network algorithm prediction values and experimental values.

Figure 15 shows the correlation between the predicted values and the experimental values in the validation set. It can be seen that most of the data fall within the mean relative error range of ±3.00%, and the *RMSE* and *R* values were reported as 7.311 and 0.997, respectively. These findings indicate that the BP neural network algorithm has greater accuracy in predicting the flow stress of the (2.5 vol%TiB + 2.5 vol%TiC)/TC4 in comparison to the Arrhenius model.

## 5. Conclusions

In this study, the stress–strain data of (2.5 vol%TiB + 2.5 vol%TiC)/TC4 under different temperatures and strain rates were obtained by hot compression experiments. Based on the experimental results, the constitutive models of the TMCs were established by the Arrhenius model and the BP neural network algorithm, respectively. The main conclusions are the following:

(1) The flow stress of the (2.5 vol%TiB + 2.5 vol%TiC)/TC4 increases with the strain rate increasing and the hot working temperature decreasing. The hot deformation process is influenced by microstructure evolution behaviors such as dynamic recovery, dynamic globularization, and dynamic recrystallization. Additionally, the stress–strain curve has obvious work hardening and softening behaviors.

(2) Based on the hot compression experimental results, constitutive models of (2.5 vol%TiB + 2.5 vol%TiC)/TC4 were established using the Arrhenius model and the BP neural network algorithm. The average absolute relative error, root mean square error, and correlation coefficient of the Arrhenius model are 6.54%, 16.769, and 0.994, respectively. The average absolute relative error, root mean square error, and correlation coefficient of the BP neural network algorithm are 3.00%, 7.311, and 0.997, respectively.

(3) Both the Arrhenius model and the BP neural network algorithm can be used to predict the flow stress of (2.5 vol%TiB + 2.5 vol%TiC)/TC4 under different hot working conditions. However, the BP neural network algorithm offers a simpler modeling process and higher prediction accuracy, making it more suitable for accurately predicting the hot compression flow stress of TMCs.

## Figures and Tables

**Table 1:** Experiment conditions.

Serial Number	Strain Rate (s^−1^)	Temperature (K)
1	0.001	1013.15
2	0.01	
3	0.1	
4	0.001	1053.15
5	0.01	
6	0.1	
7	0.001	1093.15
8	0.01	
9	0.1	
10	0.001	1133.15
11	0.01	
12	0.1	

**Table 2:** Material constants of Arrhenius model.

ε	*α*	*m*	*Q* (kJ/mol)	lnA
0.1	0.005396	3.8622	339.236	32.356
0.2	0.005514	3.5735	310.272	29.334
0.3	0.005773	3.4156	294.554	27.724
0.4	0.006166	3.2785	282.275	26.409
0.5	0.006542	3.1902	270.509	25.185
0.6	0.006927	3.1987	273.668	25.604
0.7	0.007235	3.2396	272.444	25.448

## Data Availability

The data presented in this study are available on request from the corresponding author.
